# Lentil lectin derived from *Lens culinaris* exhibit broad antiviral activities against SARS-CoV-2 variants

**DOI:** 10.1080/22221751.2021.1957720

**Published:** 2021-08-01

**Authors:** Wenbo Wang, Qianqian Li, Jiajing Wu, Yu Hu, Gang Wu, Chuanfei Yu, Kangwei Xu, Xumei Liu, Qihui Wang, Weijin Huang, Lan Wang, Youchun Wang

**Affiliations:** a Division of Monoclonal Antibody Products, National Institutes for Food and Drug Control (NIFDC), Beijing, People’s Republic of China; b Division of HIV/AIDS and Sex-Transmitted Virus Vaccines, National Institutes for Food and Drug Control (NIFDC), Beijing, People’s Republic of China; c Graduate School of Peking Union Medical College, Beijing, People’s Republic of China; d Wuhan Institute of Biological Products, Hubei, People’s Republic of China; e CAS Key Laboratory of Pathogenic Microbiology and Immunology, Institute of Microbiology, Chinese Academy of Sciences (CAS), Beijing, People’s Republic of China; f School of Life Sciences, University of Science and Technology of China, Hefei, People’s Republic of China; g Division of Respiratory Virus Vaccines, National Institutes for Food and Drug Control (NIFDC), Beijing, People’s Republic of China; h School of Pharmacy, Yantai University, Yantai, People’s Republic of China

**Keywords:** SARS-CoV-2, antiviral lectin, carbohydrate-binding protein, N-linked glycosylation, pseudovirus

## Abstract

The spike (S) protein of severe acute respiratory syndrome coronavirus 2 (SARS-CoV-2) mutated continuously and newly emerging variants escape from antibody-mediated neutralization raised great concern. S protein is heavily glycosylated and the glycosylation sites are relatively conserved, thus glycans on S protein surface could be a target for the development of anti-SARS-CoV-2 strategies against variants. Here, we collected 12 plant-derived lectins with different carbohydrate specificity and evaluated their anti-SARS-CoV-2 activity against mutant strains and epidemic variants using a pseudovirus-based neutralization assay. The *Lens culinaris*-derived lentil lectin which specifically bind to oligomannose-type glycans and GlcNAc at the non-reducing end terminus showed most potent and broad antiviral activity against a panel of mutant strains and variants, including the artificial mutants at N-/O-linked glycosylation site, natural existed amino acid mutants, as well as the epidemic variants B.1.1.7, B.1.351, and P.1. Lentil lectin also showed antiviral activity against SARS-CoV and MERS-CoV. We found lentil lectin could block the binding of ACE2 to S trimer and inhibit SARS-CoV-2 at the early steps of infection. Using structural information and determined N-glycan profile of S trimer, taking together with the carbohydrate specificity of lentil lectin, we provide a basis for the observed broad spectrum anti-SARS-CoV-2 activity. Lentil lectin showed weak haemagglutination activity at 1 mg/mL and no cytotoxicity activity, and no weight loss was found in single injection mouse experiment. This report provides the first evidence that lentil lectin strongly inhibit infection of SARS-COV-2 variants, which should provide valuable insights for developing future anti-SARS-CoV-2 strategies.

## Introduction

Coronavirus disease 2019 (COVID-19), an acute respiratory disease caused by the severe acute respiratory syndrome coronavirus 2 (SARS-CoV-2), has rapidly developed into a pandemic throughout the world. SARS-CoV-2 is a rapidly evolving RNA virus that continually accrues genomic mutations as it transmits. Although the proofreading activity of 3′-5′ exonuclease can partially correct some errors made during replication and lead the coronavirus to have a lower mutation rate compared with most other RNA viruses, a variety of SARS-CoV-2 mutations and variants have emerged during the epidemic.

SARS-CoV-2 S protein has been the major site of antigenic variation and immune escape, owing to its importance during virus entry and its ability to induce NAb responses. Indeed, mutations in the S protein that mediate evasion of antibody neutralization or enhancement of viral infectivity were identified [1–5], the D614G mutation enhanced SARS-CoV-2 infection to multiple human cells [1,2], and D614G combined mutants (D614G + I472V, D614G + A435S) decreased neutralization susceptibility to mAbs or convalescent sera [2]. Mutation K417N, E484K, and N501Y mediated enhancement of viral infectivity and/or neutralization resistance were also reported [3–5]. The recently emerged SARS-CoV-2 variants B.1.1.7 (501Y.V1), B.1.351 (501Y.V2), and P.1 (501Y.V3) containing 9–12 mutations in S protein were more resistant to mAbs (anti-RBD and/or anti-NTD antibodies), convalescent plasma and vaccinee sera [6–12]. These natural mutations and variants present challenges for mAb therapy and threaten the efficacy of vaccines, raised widespread concern.

The S-trimer protein of SARS-CoV-2 is heavily glycosylated and each monomer contains 22 N-linked glycosylation sites [6,13]. Glycosylation sites are under selective pressure as they facilitate immune evasion by shielding specific epitopes from antibody neutralization. However, we note that the N-linked glycosylation sites of SARS-CoV-2 S protein are highly conserved [6,7] and rare mutations (4 of 10333 strains) at glycosylation site were observed (N17K, T1136I, and S151I/G) [8]. Thus, antiviral strategies that aim at glycans of the virus particle can be advantageous for combating SARS-CoV-2 variation.

Lectins are proteins that bind to specific carbohydrate structures [9], and many lectins have been discovered to have antiviral activity against various viruses, including HIV, HCV, HSV-2, RSV, and influenza viruses [10–12]. Among the antiviral lectins, the well-studied Griffithsin (GRFT) which was isolated from the red algae *Griffithsia* sp. is a leading one that showed potent antiviral activity against HIV and numerous other viruses, and in clinical trials as a topical vaginal gel for the prevention of HIV [14]. Lectins inhibit SARS-CoV, MERS-CoV, and other mammalian and avian coronaviruses were also reported [15–17]. Although the glycan shield of SARS-CoV-2 S protein is consistent with other coronaviruses [6,18], differences in surface glycan pattern and glycosylation sites were existed [13,18], and the effectiveness of lectins against SARS-CoV-2 and the antigenic variants were not clear. To date, only one recent research demonstrate that a plant-derived lectin FRIL which directly binds to virus particle, has antiviral activity against a SARS-CoV-2 strain collected from Taiwan [19], the antiviral activity against SARS-CoV-2 antigenic variants were not investigated.

In the present study, we collected 12 plant-derived lectins that specific to different carbohydrate structure and evaluated their antiviral activity against a panel of SARS-CoV-2 mutant strains and epidemic variants, include the investigational mutants at N- or O-linked glycosylation site, the natural amino acid mutants and three epidemic variants. The lentil lectin which bind to both the high mannose glycan and N-terminal GlcNAc showed the most potent and broad anti-SARS-CoV-2 activity. The haemagglutination and cytotoxic activity, and mode of action of lentil lectin were also characterized.

## Materials and methods

### Lectins and reagents

Lentil lectin, wheat germ agglutinin (WGA), maackia amurensis lectin (MAL), peanut lectin, sambucus sieboldiana lectin (SSL) were purchased from Wako (Japan). The lectin from *Datura stramonium* (DSL, jimson weed, thorn apple), succinyl-concanavalin A (succ-Con A), lectin from *Galanthus nivalis* (snowdrop, GNL), erythroagglutinin PHA-E, leucoagglutinin PHA-L, phytohaemagglutinin PHA-M and phytohaemagglutinin PHA-P from *Phaseolus vulgaris* (red kidney bean) were purchased from Sigma Aldrich. The carbohydrate specificity of lectins are as follows: lentil lectin and succ-Con A specifically bind to the Man/GlcNAc/Glc [20], WGA specifically binds to the GlcNAc/Neu5Ac [20], MAL binds to the Neu5Acα3Gal [21], SSL binds to the Neu5Acα6Gal/GalNAc [20, 21], DSL binds to the Galβ3GlcNAc [22], GNL binds to manα3man [23], peanut lectin binds to Gal/GalNAc [22], PHA-E, PHA-L, PHA-M and PHA-L bind to complex-type N-glycans [24].

The SARS-COV-2 S trimer expressed in HEK293 cells were purchased from ACROBiosystems (Beijing, China).

### Cells and plasmid

293 T cells were obtained from American Type Culture Collection (ATCC), and Huh7 cells for anti-viral assay were obtained from the Japanese Collection of Research Bioresources (JCRB).

SARS-CoV-2 spike (original strain, MN908947; hCoV-19/South Africa/KRISP-K007869/2020, B.1.351 lineage, EPI_ISL_860630; hCoV-19/England/QEUH-F56F0F/2021, B.1.1.7 lineage, EPI_ISL_852526; hCoV-19/Brazil/AM-991/2020, P.1 lineage, EPI_ISL_833171), SARS-CoV spike (GenBank: AY278491), MERS-CoV spike (GenBank: AFS88936.1) and VSV glycoprotein (GenBank: M27165) expressing plasmids were constructed as described previously [2,25]. The spike expressing plasmid of the original strain was used as the template for mutagenesis.

### Pseudovirus preparation, titration and anti-viral assays

Pseudovirus preparation, titration, and pseudovirus-based anti-viral assay were performed as described previously [2,25]. Pseudovirus-based anti-viral assay was measured as a reduction in luciferase expression after a single-round infection of Huh7 cells. Briefly, 100 μL serial dilutions of lectin preparations were added into 96-well plates, then 50 μL pseudoviruses (1300 TCID_50_/mL) were added and incubated at 37°C for 1 h. A 100 μL Huh7 cells (2 × 10^5^ cells/mL) were then added and incubated at 37°C in a humidified atmosphere with 5% CO_2_. Chemiluminescence detection was performed after 24 h incubation. The Reed-Muench method was used to calculate the IC_50_ of each lectin.

### Authentic SARS-CoV-2 neutralization CPE assay

Neutralization of authentic SARS-CoV-2 was performed using a cytopathic effect (CPE) assay. Briefly, serial diluted lentil lectin were prepared in a 96-well tissue culture plate and equal volume of authentic SARS-CoV-2 virus (hCoV-19/China/CAS-B001/2020 strain, National Microbiology Data Center: NMDCN0000102 and GISAID databases: EPI_ISL_514257, isolated and identified by Dr. Yuhai Bi.) containing 100 TCID_50_ was added, then incubated at 37°C for 1 h. The lectin – virus mixture was then transferred into a 96-well plate containing an equal volume of confluent Vero E6 cells with eight repeats and incubated at 37°C for three days. The CPE in each well was observed on day 3 after infection. All experiments were performed in a Biosafety Level 3 facility.

### Cytotoxicity testing

The 50% cytotoxic concentration (CC_50_) of lectins were determined by CellTiter-Glo luminescent cell viability assay kit (Promega, Madison, WI). Specifically, serial dilutions of lectins starting from 1 mg/mL were mixed with Huh7 or 293T cells in 96-well plates and incubated at 37°C for 24 h, the cell viability was analysed using a microplate luminometer (Promega, Madison, WI). The CC_50_ was determined by the dose–response curve using nonlinear regression.

### SEC-MALS

Size exclusion chromatography was performed by running the lectin through TSK G3000 SWXL column (Tosoh), on an HPLC connected to a three-angle light-scattering detector (DAWN) and a refractive index detector (Optilab T-rEX, Wyatt Technology). Data analysis was done with ASTRA 7.

### Analysis of binding activity of lectins to SARS-CoV-2 S protein by SPR

SPR were carried out on the T200 instruments (BIAcore, Cytiva). S trimer were immobilized on the second flow cell of a CM5 chip using amine coupling. Briefly, the sensor chip surface was activated by the 1:1 mixture of 400 mM Nethyl-N-(3-dimethylaminopropyl)-carbodiimide hydrochloride and 100 mM N-hydroxysuccinimide, and then S trimer (25 μg/mL in 10 mM sodium acetate, pH5.0) were injected over the activated surface to reach about 1000 response units (RU). The residual reactive surface was blocked at 10 μL/min for 7 min by 1.0 M ethanolamine/HCl, pH 8.5. Flow cell 1 was treated similarly but without injection of any ligand and used as a blank control.

Lectins were serially diluted in sample buffer (0.01 M HEPES, 0.05% polysorbate 20, 150 mM Nacl and 3 mM EDTA) and injected over the chip at 30 μL/min for 2 min, then dissociated for 150 s. The surfaces were regenerated with glycine 1.5 (Cytiva) at 20 μL/min for 30 s to remove the bound lectins.

### Haemagglutination assays

Red blood cells of the rooster were washed and resuspended at a final concentration of 1% (v/v) in PBS. A 50 μL 2-fold serial diluted lectins were mixed with an equal volume of erythrocytes in a 96-well round-bottom plate. The influenza antigen (B/Maryland/15/2016, NIBSC-UK-EN63QG, NIBSC code:18/104, HA: 69 µg/mL) was 2-fold serial diluted and performed as a positive control. The plate was incubated for 1 h at RT and haemagglutination activity was determined by visual examination.

### Glycan array analysis

Lentil lectin was labelled with Cy3 and its bioactivity was checked using pseudovirus-based anti-viral assay. N-Glycan microarray slides (Creative Biochip, Nanjing, China) were blocked for 30 min with blocking buffer and then washed with PBST (PBS buffer, 0.05% Tween 20). 1–8 μg/mL lectin-Cy3 was added to the array and incubated at 37°C for 2 h, then washed to remove unbound lectins. Microarray slides were then scanned and analysed.

### SPR-based competitive binding assay

The competitive assay of lentil lectin on ACE2-S trimer binding was analysed using the BIAcore T200 system (BIAcore, Cytiva) at 25°C. Fc-tagged human ACE2 were captured onto the second flow cell of series S sensor chip CM5 via anti-human IgG (Fc) antibody (Cytiva) to yield a response of 350 RU. A 400 nM SARS-CoV-2 S protein were prepared in the running buffer (1× HEPES with 0.005% Tween-20) containing various concentration of lentil lectin (0, 0.5, 5, 20, and 50 nM), then incubated at room temperature for 10 min and run over ACE2. Response to human ACE2 binding was measured at a flow rate of 30 μL/min with an association time of 60 s and dissociation time of 90 s. Binding kinetic parameters were evaluated with Biacore® Insight software (BIAcore, Cytiva).

### Peptide mapping and glycopeptide analysis by mass spectrometry

To identify the amino acid sequence, 100 μg lentil lectin were denatured in 50 mM Tris/HCl (pH 7.5) containing 6 M Gdn-HCl. Next, the lectin were reduced and alkylated by adding DTT and iodoacetamide (IAM) respectively, followed by a 1 h incubation with 20 mM DTT to eliminate residual IAM. Then, the proteins were buffer-exchanged into 50 mM Tris/HCl (pH 7.5) using NAP-5 Columns and digested at 37°C for 4 h by trypsin or chymotrypsin (Promega) at a ratio of 1:20 (w/w). Peptides were separated using an ACQUITY UPLC BEH C18 column (2.1 mm × 150 mm) and analysed by Q Exactive Plus system (Thermo Fisher Scientific). The spray voltage was set to 3.8 kV and the capillary temperature was set to 280°C. The scan range was 200−2000 m/z.

To analyse the site-specific N-glycan profile, 100 μg of SARS-CoV-2 S protein were denatured, reduced, alkylated, and digested as the details mentioned above, then analysed by nanoLC-ESI MS with an U3000 nano RSLC (Thermo Fisher Scientific) system coupled to a Orbitrap Eclipse mass spectrometer (Thermo Fisher Scientific) using higher energy collision-induced dissociation (HCD) fragmentation. Peptides were separated using an Acclaim PepMap 100 C18 column (75 µm × 25 cm). The spray voltage was set to 2.1 kV and the ion transfer tube temperature was set to 320°C. The HCD collision energy was set to 30%, and scan range was 350−2000 m/z. Precursor and fragment detection were performed using Orbitrap at a resolution MS1 = 60,000, MS2 = 30,000. The AGC target for MS1 = 4e5 and MS2 = 5e4, and injection time for MS1 = 50 ms, MS2 = 75 ms. Glycopeptide fragmentation data were extracted from the raw file using Byonic™ software (Version 3.8; Protein Metrics Inc.) in Proteome Discoverer (Version 2.5; Thermofisher).

### Statistical analysis

Error bars indicate the standard deviation of the means calculated by Prism software 5.0. One-way analysis of variance with Tukey’s multiple comparison was used to test whether the mice bodyweight is significantly different between lentil lectin- and PBS-treatment by SPSS 24.0. A *p* value <.05 was considered significant and is depicted by an asterisk in Supplementary Figure 3.

## Results

### Evaluation of binding activity of lectins to SARS-CoV-2 S protein and their antiviral activity

Twelve plant lectins from different species were chosen to evaluate their antiviral activity against SARS-CoV-2. Firstly, we determined whether these lectins could directly interact with SARS-CoV-2 S protein using a surface plasmon resonance (SPR) method. The molecular weight (Mw) of each lectin determined by SEC-MALS was used to calculate the KD value. As shown in Table 1, except for peanut lectin and MAL, which showed no detectable binding activity, other lectins could bind to S protein with different affinities. The WGA, DSL, and GNL showed the most potent binding activity, where the succ-Con A was the lowest.

**Table 1.** Lectins tested for antiviral activity against SARS-CoV-2 pseudovirus.

Lectin	Mw (kD)	ka (1/Ms)	kd (1/s)	KD (M)	IC_50_ (μg/mL)	haemagglutination activity (μg/mL)	CC_50_ (μg/mL)	SI
WGA	38.4	3.21E+06	4.82E-03	1.50E-09	8.5 ± 0.6	>7.81	>500	>58.8
SSL	133.7	2.81E+04	8.86E-04	3.15E-08	73.6 ± 8.1	>0.98	>500	>6.8
Lentil lectin	52.5	1.06E+05	9.19E-04	8.67E-09	8.9 ± 0.9	>500	>500	>56.2
Peanut lectin	98.8	na	na	na	>100	>1000	>500	na
MAL	122.1	na	na	na	>100	>7.81	>500	na
PHA-L	109.2	6.97E+04	2.62E-03	3.76E-08	22.0 ± 1.8	>3.91	>500	>22.7
PHA-P	150.8	1.54E+05	5.01E-03	3.25E-08	75.2 ± 5.2	>31.25	>500	>6.6
PHA-M	118.1	7.89E+03	1.96E-02	2.48E-06	53.8 ± 10.3	>31.25	>500	>9.3
PHA-E	118.4	2.07E+04	3.31E-03	1.60E-07	20.2 ± 3.5	>3.91	>500	>24.8
DSL	68.4	1.75E+06	1.44E-03	8.23E-10	84.0 ± 10.0	>0.49	>500	>6.0
succ-Con A	51.2	7.37E+00	1.48E-03	2.01E-04	33.8 ± 3.9	>1000	>500	>14.8
GNL	40.3	7.92E+05	1.21E-03	1.53E-09	72.0 ± 3.4	>0.5	>500	>6.9

**Figure 1.**
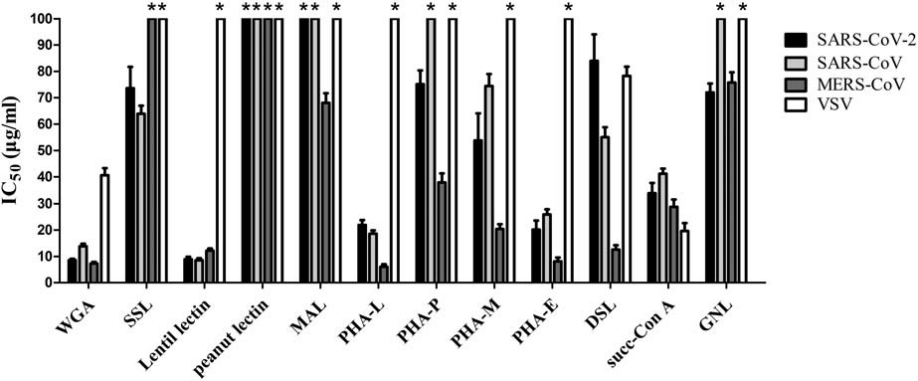
Antiviral activity of lectins against SARS-CoV-2, SARS-CoV, MERS-CoV, and VSV by pseudovirus-based neutralization assay. Data are from three independent experiments, and the error bars indicate the SDs from the mean values. *, pseudovirus could not be neutralized by lectins at the highest concentration (100 μg/mL).

Next, a pseudovirus-based neutralization assay performed on Huh7 cells was used to investigate whether the binding of S protein could mediate viral inhibition against SARS-CoV-2 (original strain), and the SARS-CoV/MERS-CoV S protein and VSV glycoprotein bearing pseudoviruses were also tested. Preincubation of pseudoviruses with lectins showed a different reduction of viral infection. The WGA, lentil lectin, PHA-L, and PHA-E showed most potent antiviral activity against SARS-CoV-2 pseudovirus with IC_50_ range from 8.5 to 22.0 μg/mL (Table 1 and Figure 1) and also inhibit infection of both SARS-CoV and MERS-CoV pseudovirus. It is of note that among the four potent lectins, only WGA inhibit the VSV (IC_50_: 40 μg/mL). Other tested lectins also showed antiviral activity at higher IC_50_. Succ-Con A and DSL inhibit all four pseudoviruses, SSL inhibit both SARS-CoV and SARS-CoV-2 but not MERS-CoV (Figure 1). The peanut lectin and MAL which showed no binding activity to SARS-CoV-2 S protein, also had no antiviral activity against SARS-CoV-2. We also checked the antiviral activities of the twelve lectins against SARS-CoV-2 (original strain) using 293 cells stably expressing the ACE2 receptor (293-ACE2) and Vero cells, the IC_50_ values obtained from 293-ACE2 and Vero cells were basically comparable to Huh7 cells, and the WGA, lentil lectin, PHA-L and PHA-E were still the most potent lectins where peanut lectin and MAL showed no antiviral activity (data not shown).

### Inhibition of the infection of SARS-CoV-2 S mutant strains and epidemic variants by lectins

Ten of the 12 lectins (except for peanut lectin and MAL) we tested showed inhibitory effect on SARS-CoV-2 pseudovirus (original strain) infection, next we evaluated their antiviral activity against a panel of mutant strains and variants using a pseudovirus-based neutralization assay, including the artificial mutants at N-/O-linked glycosylation site, natural existed amino acid mutants which had an impact on antigenicity or showed increased mutation frequency, as well as the concerned variants B.1.1.7, B.1.351, and P.1.

**Figure 2.**
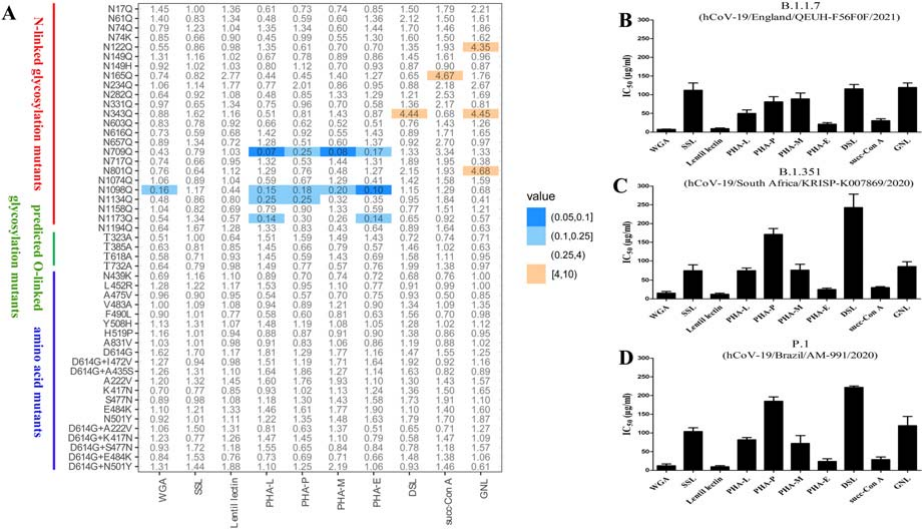
Antiviral activity of lectins against mutant strains and epidemic variants by pseudovirus-based neutralization assay. (A) Neutralization susceptibility of mutant strains to lectins. The fold difference of IC_50_ is present by different colors, i.e. under 4-fold change in white. Fold difference was calculated by dividing the mean IC_50_ (mutant) by mean IC_50_ (WT). Antiviral activity of lectins to three epidemic variants B.1.1.7 (B), B.1.351 (C) and P.1 (D) were determined. Data are from three independent experiments, and the error bars indicate the SDs from the mean values.

Lectins target the sugar moieties of glycoprotein. Firstly, we investigate whether the variations of glycosylation site on SARS-CoV-2 S protein could impact the neutralization susceptibility to lectins. Totally 28 artificial mutants with deletion of N- (22 sites, 24 mutants) or O-linked (4 predicted sites, 4 mutants) glycosylation site were constructed and evaluated. As shown in Figure 2(A), the antiviral activity of SSL and lentil lectin to all these 28 glycosylation mutants were not affected with the fold change of IC_50_ was under 4.0. We found some glycosylation site mutants showed reduced susceptibility to lectins, where N122Q and N801Q had 4-fold reduced susceptibility to GNL, N165Q had 4-fold reduced susceptibility to succ-Con A, and N343Q had 4-fold reduced susceptibility to both DSL and GNL (Figure 2(A)). Interestingly, elimination of N-linked glycosylation site on S2 subunit increased susceptibility to some lectins. Mutation N709Q, and three mutations located at the membrane-proximal C terminus (N1098Q, N1134Q, and N1173Q) had 4–10 fold increase in susceptibility to PHA-L, PHA-P, PHA-M, and PHA-E. N1098Q also had a 6-fold increase in susceptibility to WGA.

In a previous study, we identified several natural mutants with decreased sensitivity to neutralizing mAbs or convalescent sera, include D614G, the D614G combined mutants (D614G + I472V, D614G + A435S) and other single amino acid mutants [2]. Next, we investigated the antiviral activity of lectins to these mutants, and the recently reported mutants (A222V [26], K417N, S477N, E484K, and N501Y or combined with D614G, respectively) and three epidemic variants (British strain B.1.1.7, South African strain B.1.351, and Brazil strain P.1) were also included. As expected, to these mutants that maintained the entire glycosylation sites, the antiviral activity of lectins were not influenced with a fold change of IC_50_ was under 4.0 compared with the original strain (Figure 2(A)). We also found that most lectins showed similar antiviral activity to the three epidemic variants (B.1.1.7, B.1.351, and P.1) compared with the original strain, and a 2-fold higher IC_50_ of PHA-L, PHA-P, and DSL were found to epidemic variants B.1.351 and P.1 (Figure 2(B–D)).

### Haemagglutination and cytotoxicity activity of lectins

Since many natural lectins cause haemagglutination, we investigated the haemagglutination activity of lectins to blood samples from rooster, and the cytotoxic activity to Huh7 and 293T cells were also investigated. As expected, the PBS did not show any haemagglutination activity, and the known haemagglutinating agent influenza HA demonstrated activity at concentrations above 0.03 μg/mL (Supplementary Figure 1). Most of the tested lectins showed haemagglutination activity, and the DSL had the strongest haemagglutination activity which the erythrocytes were affected at 0.49 μg/mL (Table 1 and Supplementary Figure 1). The SSL showed haemagglutination activity at 0.98 μg/mL, PHA-L and PHA-E at 3.91 μg/mL, WGA and MAL at 7.81 μg/mL, PHA-M and PHA-P at 31.25 μg/mL, and GNL at 0.5 mg/mL. The lentil lectin showed some weak haemagglutination activity at the highest concentrations tested (1 mg/mL). The peanut lectin which showed no binding to S protein and no antiviral activity also had no haemagglutination activity at 1 mg/mL. Succ-Con A, the succinylated Con A, which was reported that the agglutination activity is greatly reduced by the succinylation [27], showed no haemagglutination activity at 1 mg/mL.

The cytotoxicity activity of lectins to both Huh7 and 293T cells, represented by CC_50_ (50% cytotoxic concentration) are shown in Table 1. All lectins showed no cytotoxicity at 500 μg/mL.

Using pseudovirus-based neutralization assay, we identified several lectins that showed potent and broad antiviral activity. Among them, WGA and lentil lectin showed the most potent antiviral activity against SARS-CoV, SARS-CoV-2 and MERS-CoV, and also a broad variety of SARS-CoV-2 variants. Considering that the WGA had a strong haemagglutination activity at 7.81 μg/mL where the lentil lectin only had weak haemagglutination activity at 1 mg/mL, the lentil lectin was more suitable to be a candidate as SARS-CoV-2 inhibitor and its mode of action was further studied.

### Mechanistic analyses of lentil lectin against SARS-CoV-2 infection

**Figure 3.**
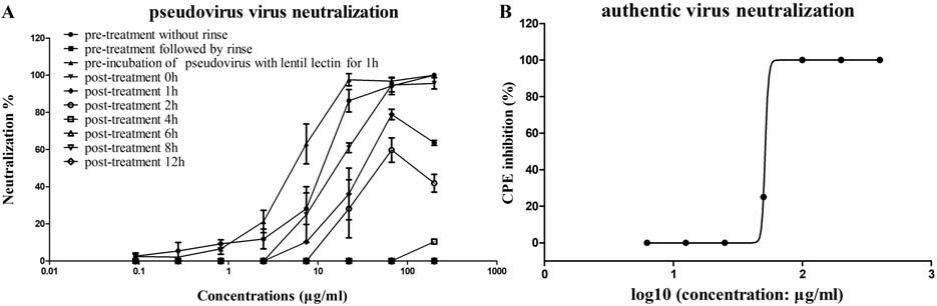
Lentil lectin inhibit SARS-CoV-2 infection at early steps. (A) Inhibition of pseudovirus infection by lentil lectin with pre- and post-treatment. (B) Authentic SARS-CoV-2 neutralization potency measured by CPE Assay. Data are from three independent experiments, and the error bars indicate the SDs from the mean values.

The lentil lectin used in the current study were derived from *Lens culinaris* and the amino acid sequence was highly similar to the reported sequence Q8VXF2.2 (only one amino acid difference at position 89, Supplementary Figure 2). To investigate how lentil lectin inhibit SARS-CoV-2 infection, we studied its mode of action *in vitro*. First, the lentil lectin were pre- and post-treated to Huh7 cells or pseudoviruses and evaluated its antiviral activity. For lentil lectin pre-treatment, Huh7 cells were pre-treated with serial diluted lectins at 37°C for 1 h, after five times rinse by PBS or with no rinse, the cells were then infected with SARS-CoV-2 pseudovirus. For post-treatment, Huh7 cells were infected with pseudovirus at 37°C for 0, 1, 2, 4, 6, 8 or 24 h, and then treated with lentil lectin at different concentrations. Marked inhibitory effects on viral infectivity were observed in post-treatment at 0, 1, and 2 h and pre-incubation of pseudovirus with lentil lectin for 1 h (Figure 3(A)). In addition, pre-incubation of Huh7 cells with lentil lectin followed extensively PBS rinse of cells to eliminate residual lentins showed no inhibition where the pre-incubation with no PBS rinse resulted in significant dose-dependent inhibition of infectivity. Furthermore, the inhibitory effect decreased as the later time of lentil lectin addition in post-treatment, and no viral inhibition was found after 4 h post-treatment. Thus, lentil lectin may acted at the early steps of viral infection, and react directly with SARS-CoV-2 but not the receptors on Huh7 cells (also not react with receptors on 293-ACE2 cells and VERO cells, data not shown). In an authentic virus-based CPE inhibition assay, lentil lectin preincubated with live infectious SARS-CoV-2 showed robust inhibition with IC_50_ at 60.26 μg/mL (1.2 μM) (Figure 3(B)).

**Figure 4.**
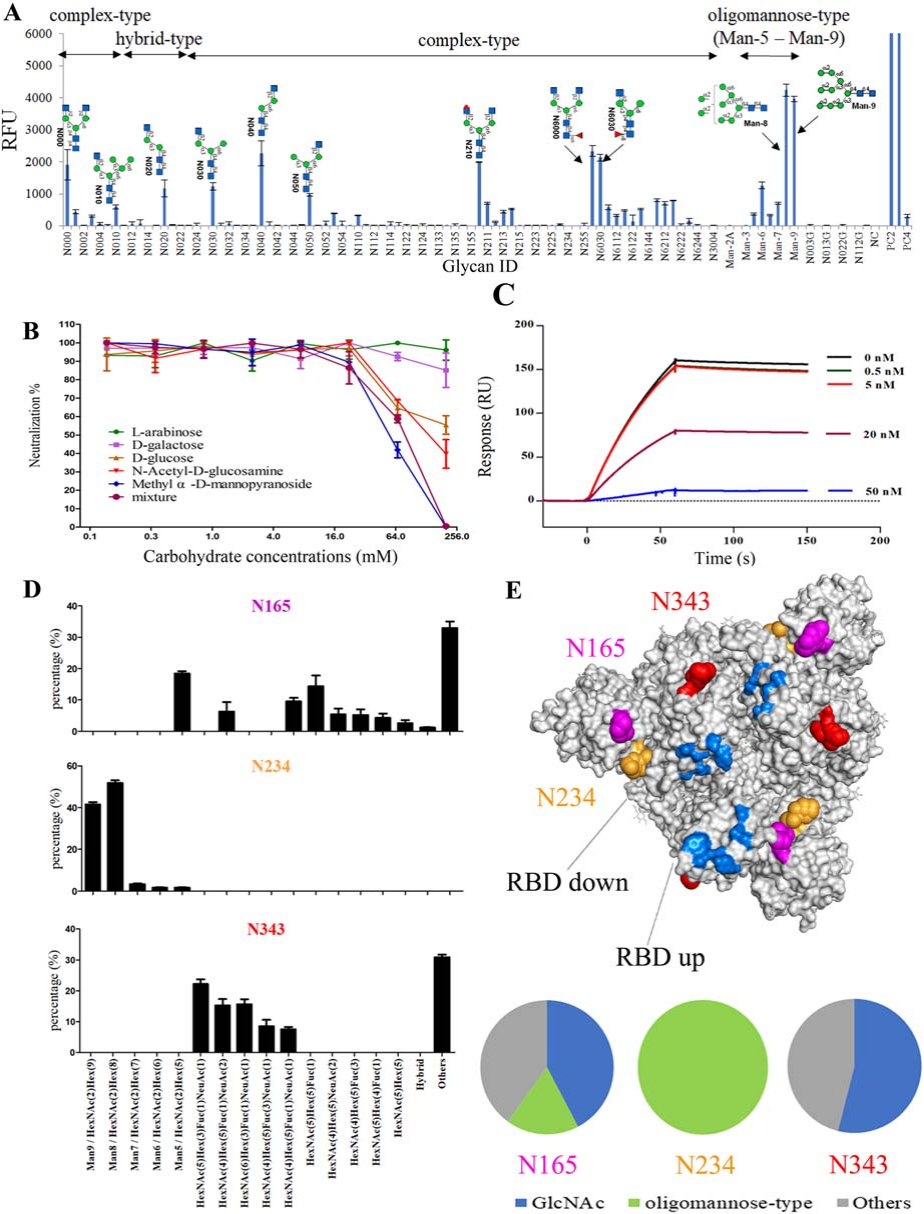
Carbohydrate specificity of lentil lectin and its reaction to SARS-CoV-2 S protein. (A) Glycan array with Cy3-labelled lentil lectin. Symbol nomenclature for glycans is used to represent oligosaccharides on the graph (blue squares for GlcNAc, green circles for mannose, red triangles for fucose, and red circles for peracetylation). (B) Competitive inhibition of lentil lectin neutralization activity against SARS-CoV-2 pseudovirus by monosaccharides L-arabinose, D-galactose, D-glucose, N-Acetyl-D-glucosamine (D-GlcNAc), methyl α-D-mannopyranoside and equimolar mixture of D-GlcNAc and methyl α-D-mannopyranoside. (C) Competitive inhibition of lentil lectin on ACE2-S trimer binding. S trimer (400 nM) was incubated with various concentration of lentil lectin at room temperature for 10 min and then run over ACE2. (D) N-glycan profile of N165, N234, and N343. (E) Representative glycans at N165 (purple), N234 (yellow) and N343 (red) onto the prefusion structure of the trimeric SARS-CoV-2 S glycoprotein (original strain, GenBank: MN908947, PDB ID 6VSB) [28] with one RBD in the “up” conformation and the other two RBDs in the “down” conformation. The ACE2 receptor-binding site was indicated in light blue. The proportion of N-glycans which could bind to lentil lectin at each site was showed as a pie chart drawing according to data from panel D. The PyMOL (Version 2.4.1, Schrodinger, LLC.) was used for visualization.

We next determined the carbohydrate-binding specificity of lentil lectin using a fluorescent dye Cy3-labelled glycan array-based assay. The lentil lectin demonstrated the best binding to two subtypes of N-glycans: the oligomannose-type N-glycans ranging from Man-5 to Man-9, and N-glycans containing GlcNAc at the non-reducing end terminus (Figure 4(A)). The oligomannose-type N-glycan Man-8 and Man-9 showed the highest binding affinity and N-glycans containing GlcNAc at the non-reducing end terminus could be either monoantennary complex N-glycans (N020), biantennary complex N-glycans (N000) or the hybrid N-glycans (N010). To confirm whether the antiviral activity of lentil lectin was attributed to the binding of oligomannose-type glycans and GlcNAc on SARS-CoV-2 S protein, 100 μg/mL lentil lectin were pre-incubated with serial diluted five types of monosaccharide at 37°C for 1 h and then evaluated their anti-SARS-CoV-2 activity. As shown in Figure 4(B), pre-incubated with methyl α-D-mannopyranoside, N-Acetyl-D-glucosamine (D-GlcNAc), and D-glucose inhibit the antiviral activity of lentil lectin where the α-D-mannopyranoside showed the most potent inhibition. These data suggest that the antiviral activity of lentil lectin is directly dependent on its oligomannose-type/GlcNAc-binding function.

Lentil lectin inhibit SARS-CoV-2 at the early steps of viral infection and using an SPR-based competitive binding assay, we found lentil lectin could efficiently block the ACE2-S trimer binding at 50 nM which directly against the initial viral infection (Figure 4(C)). The glycosylation sites N165, N234, and N343 were located around the receptor-binding sites, especially when the receptor-binding domain is in the ‘down’ conformation [28]. Next, we determined the site-specific glycosylation of these three sites by mass spectrometry (Figure 4(D)). N-glycans at N234 were oligomannose-type which consists of Man-8 (53.2%), Man-9 (40.3%), Man-7 (3.4%), Man-6 (1.5%), and Man-5 (1.5%). For N165 and N343, the majority of N-glycans were complex-type, and ∼20% oligomannose-type glycan Man-5 and a small amount of hybrid-type glycans were also found at N165. The proportion of lentil lectin-specific binding glycans at each site were also analysed. Glycans at N234 were all lentil lectin preferred oligomannose-type glycan, and over half of the glycans at N165 and N343 could bind to lentil lectin (Figure 4(E)). These data support that lentil lectin could bind to N-glycans at these sites and block the binding of RBD to ACE2, resulting in the inhibition of SARS-CoV-2 infection.

## Discussion

Glycan shield of viral surface glycoprotein is involved in viral evolution [29–31] and could mask the immunogenic epitopes and protect the virus from antibody neutralization [32–34]. However, surface glycans could also be the target of antibodies and glycan-binding agents. Several broad and potent neutralizing mAbs target the specific glycan of viral surface glycoproteins were identified [35–37], and glycan-binding proteins such as lectins exhibit antiviral activities [11]. Although the highly glycosylated SARS-CoV-2 S protein is continuously mutated and antigenic variants resistant to mAbs and vaccine-generated antibodies emerged, the glycosylation sites are conserved so far, which provide a candidate target for anti-SARS-CoV-2 strategy against variants.

In the present study, we collected 12 plant-derived lectins that are specific to different carbohydrate structures and evaluated their anti-SARS-CoV-2 activity. Out of 12 lectins evaluated, 10 inhibit SARS-CoV-2 pseudovirus infection and among them, lentil lectin derived from *Lens culinaris* showed the most potent and broad antiviral activity with very weak haemagglutination activity and no cytotoxicity. The lentil lectin exhibit broad anti-SARS-CoV-2 activity against a panel of SARS-CoV-2 mutant strains and epidemic variants, including the concerned B.1.1.7, B.1.351, and P.1. The lentil lectin also exhibit antiviral activity against SARS-CoV and MERS-CoV but not VSV.

It is of note that elimination of individual N- or O-linked glycosylation site on SARS-CoV-2 S protein had no influence on neutralization susceptibility to lentil lectin, suggesting that lentil lectin may bind to glycans at multiple sites on S trimer. Indeed, a previous study determined that all 22 N-linked glycosylation sites of S protein were glycosylated and the complex- and hybrid-type glycans comprise 71% and oligomannose-type glycans comprise 28% of total N-glycans [6]. As most lectins that have antiviral activities interact predominantly with oligomannose-type glycans [11], our glycan array results indicated that lentil lectin showed strong binding to both oligomannose-type glycans (Man-5 to Man-9), and N-glycans containing GlcNAc at the non-reducing end terminus, including both the complex- and hybrid-type glycans. The carbohydrate specificity of lentil lectin combined with the N-glycan profile of S protein, could efficiently support the binding between lentil lectin and SARS-CoV-2 S protein, and may inhibit the virus by obstructing virus entry into host cells or membrane fusion in endosome.

In the time of addition assay, we found lentil lectin inhibit SARS-CoV-2 at the early steps of infection, and our SPR-based competitive binding assay indicated that lentil lectin could efficiently block the ACE2-S trimer binding. Glycosylation sites at N165, N234, and N343 were located around the RBD, and the majority of glycans at these three sites are lentil lectin-binding glycans, especially the glycans at N234 are totally oligomannose-type which could be efficiently bound by lentil lectin. Lentil lectin bind to glycans at N165, N234, and N343 may prevent the ACE2-RBD binding, which mediate the entry of SARS-CoV-2 into target cells. Interestingly, removal of any glycosylation site at N165, N234, and N343 had no effect on neutralization susceptibility to lentil lectin, suggesting that the existence of two of these glycosylation sites could support neutralization by lentil lectin. Despite mutations and antigenic variants emerged, glycosylation sites at N165, N234, and N343 were 100% conserved so far, and our previous study found that pseudovirus with two glycosylation sites elimination in RBD was non-infectious, revealing the importance of glycosylation in RBD for viral infectivity [2], whether variants with loss of two or all these three glycosylation sites will emerge and could still be neutralized by lentil lectin need further investigation. Furthermore, the unique oligomannose-type glycan profile at N234 located around RBD may also be a potential target to develop anti-SARS-CoV-2 entry inhibitors. Whether the membrane fusion in the endosome could be inhibited by lentil lectin needs to be further investigated.

Although the IC_50_ value of lentil lectin against SARS-CoV-2 was higher compared to some reported mAbs, it exhibited a broad antiviral activity against the mutant strains and epidemic variants which were resistant to antibody-mediated neutralization. In addition, lentil lectin may have advantages in the potential antibody-dependent enhancement mediated by Fcγ receptors, which is also a risk for clinical use of antibody-based vaccines and therapeutics [38–41]. As a heterologous protein, the toxicity, immunogenicity, and bioavailability related to pharmacology are hurdles for the clinical use of lectins. Despite lentil lectin showed weak haemagglutination activity at 1 mg/mL and no cytotoxicity at 500 μg/mL, and no body weight loss of Balb/c mice was found in a 20 mg/kg injection experiment (Supplementary Figure 3), more details of such properties need to be further investigated.

Several O-linked glycosylation sites of SARS-CoV-2 S protein were identified [6,42,43], and some of them may affect the virus affinity to ACE2 and viral infectivity, such as T323 with core-1 O-glycans and S494 [42,44]. In the present study, peanut lectin specifically binds to Galβ3GalNAc (core 1 structure of O-glycan) had no antiviral activity against SARS-CoV-2, and SSL which binds to α2, 6-linked sialic acid and terminal GalNAc of O-glycan, inhibit SARS-CoV-2 with higher IC_50_, and the four O-linked glycosylation site mutants had no effect on antiviral activity of SSL. More lectins specific to different O-glycan structures and additional O-linked glycosylation sites need further investigation to support the development of potential O-glycan-specific antiviral lectins against SARS-CoV-2.

Glycans on SARS-CoV-2 S protein play important roles in the viral life cycle, immune evasion, and cell infection. Studies indicated that glycans located at or around RBD may critical to the interaction with ACE2 and viral infection [2,42,44,45]. Besides the ACE2 receptor, S protein may exploit additional receptors for infection, such as TLRs, CLRs, NRP1, and GRP78, which recognize the carbohydrate moieties clustered on S protein [46]. Get more detailed information of the glycosylation, and interaction with receptors is a premise to better understand the infection and facilitate the development of glycan-binding inhibitors [47].

In summary, this study provides the first evidence that the lentil lectin from *Lens culinaris* strongly inhibit infection of SARS-CoV-2. We found that lentil lectin specifically bind to oligomannose-type glycans and GlcNAc at non-reducing end terminus and have potent anti-SARS-CoV-2 activity against mutant strains and epidemic variants. Further studies in live animals will be necessary to develop a novel antiviral strategy for lentil lectin therapy. Our findings on the selective and anti-SARS-CoV-2 activity of lectins should also trigger research on the discovery of other carbohydrate-binding antiviral agents.

## Supplementary Material

Supplementary_Figures.doc
