# Supplementary material for: Lentil lectin derived from Lens culinaris exhibit broad antiviral activities against SARS-CoV-2 variants
Source: Emerg Microbes Infect. 2021 Aug 1;10(1):1519–29. doi: 10.1080/22221751.2021.1957720 (PMC8330776; doi:10.1080/22221751.2021.1957720)
Supplement: Supplementary_Figures.doc [file TEMI_A_1957720_SM1761.doc]

**

**

**Supplementary Figure 1. Hemagglutination activity of tested lectins.** A wide range of lectin concentrations were used to examine the potential hemagglutination activity on erythrocytes from rooster. The influenza antigen (B/Maryland/15/2016, NIBSC-UK-EN63QG, NIBSC code:18/104, HA: 69 µg/mL) and PBS were used as positive and negative controls, respectively.


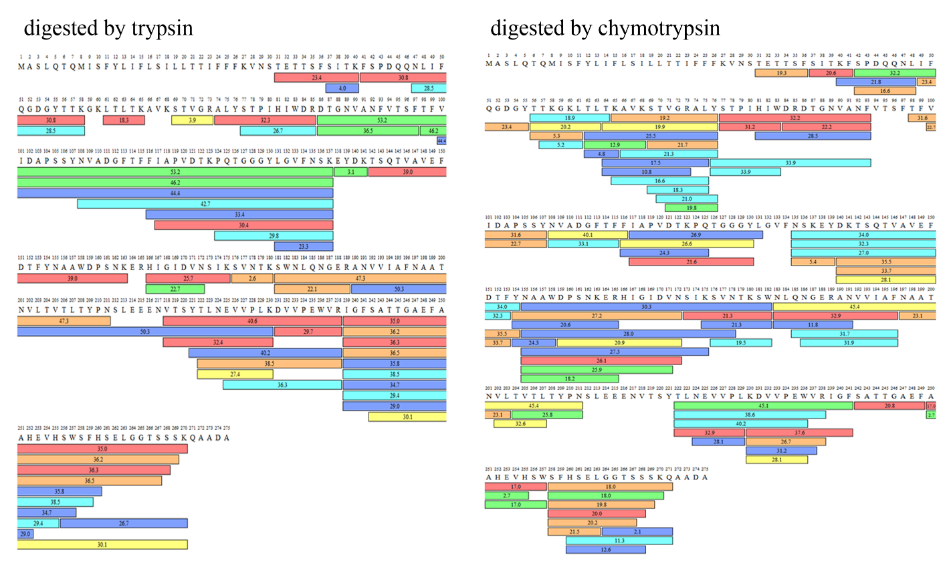


**Supplementary Figure 2. Sequence coverage map of lentil lectin by peptide mapping.** Lentil lectin was digested by trypsin or chymotrypsin and the amino acid (aa) sequence was analyzed by mass spectrometry using the sequence of lectin from *Lens culinaris* subsp. *Tomentosus* as template (Genbank: Q8VXF2.2). The N-terminal sequence containing 30 aa is signal peptide, and aa from 270 to 275 of C-terminal is propeptide. The aa sequence of lentil lectin used in the present study was identical with Q8VXF2.2 except for one aa at position 89, where lentil lectin was N89 and Q8VXF2.2 was S89.


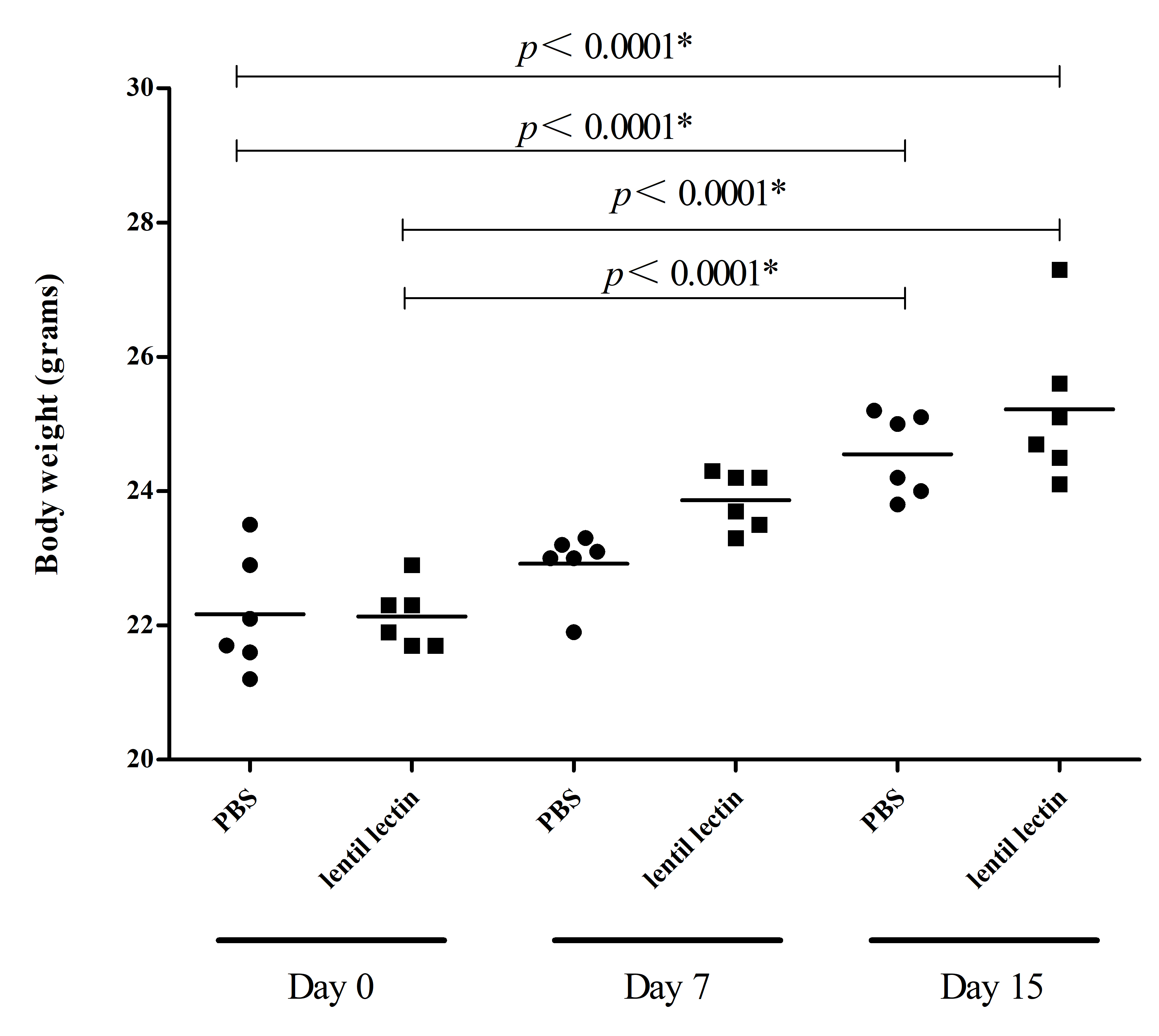


**Supplementary Figure 3. Balb/c mouse body weight gain as an indicator of overall health.** Mice were injected intraperitoneally with 20mg/kg lentil lectin (*n*=6) or PBS (*n*=6) at day 0, and body weights were measured daily post injection. A *p* value <0.05 was considered significant and depicted by an asterisk. No significant weight difference were found between PBS and lentil lectin injected mice group at the same day.
